# Epidemiological characteristics of bovine besnoitiosis (*Besnoitia besnoiti*) in a beef cattle farm: a cross-sectional serological assessment

**DOI:** 10.3389/fvets.2023.1158235

**Published:** 2023-04-26

**Authors:** Joana Coelho, Joana Domingues, Helga Waap, George Stilwell

**Affiliations:** ^1^Animal Behaviour and Welfare Laboratory, Centre of Interdisciplinary Research in Animal Health, Faculty of Veterinary Medicine, Lisbon University, Lisbon, Portugal; ^2^Associate Laboratory for Animal and Veterinary Sciences (AL4AnimalS), Lisbon, Portugal; ^3^Laboratório de Parasitologia, Instituto Nacional de Investigação Agrária e Veterinária, Oeiras, Portugal

**Keywords:** bovine besnoitiosis, *Besnoitia besnoiti*, emerging diseases, beef cattle, antibody prevalence, indirect fluorescein antibody test, risk factors

## Abstract

**Background:**

Bovine besnoitiosis is an emerging disease caused by the protozoa *Besnoitia besnoiti* that can have a serious economic impact on affected farms. The fact that there is no effective vaccine nor treatment, along with the lack of consistent epidemiologic data, renders the implementation of preventive medicine and control strategies much harder.

**Objectives:**

A cross-sectional serological assessment was performed to better understand the distribution and prevalence of this parasite in a large beef cattle farm in Portugal and to establish some epidemiological characteristics of besnoitiosis.

**Methods:**

A random blood sampling of 450 animals from a farm that keeps around 2,000 cattle head was performed and sera were submitted to an indirect immunofluorescent antibody test (IFAT). Data on breed, age, sex, and birthplace of the tested animals and their mothers were recorded.

**Results:**

The overall prevalence of positive animals was 16.89%, with significant differences between under 1-year-old calves (4.8%) and adults (19.67%). A higher antibody prevalence was shown in animals 1–2 years and >7 years old, in Salers breed and in cows imported from France or whose mothers had come from this country. Calves under 1 year old and crossbreed animals with ancestry born in the current farm presented the lowest antibody prevalence.

**Discussion and conclusions:**

The most significant risk factors revealed were age (>7 years old) and breed (Salers). Genetic studies should be carried out in order to confirm whether indeed there is a breed susceptibility to bovine besnoitiosis. We suggest that similar studies should be performed across southern Europe to establish strong epidemiologic data that would allow a rigorous transnational control program to be launched.

## 1. Introduction

Bovine besnoitiosis is an emerging disease (1) resulting from the infection by *Besnoitia besnoiti*, an obligate intracellular protozoan that has tropism for skin and connective tissue. Mortality rate is relatively low (up to 10%), but morbidity is usually high in affected herds (2). Weight loss, anasarca, hyperkeratosis, abortions, and necrotizing orchitis, leading to transient infertility or even sterility, characterize clinical cases of bovine besnoitiosis, resulting in severe economic and welfare consequences (3, 4).

Domestic and wild bovid as well as cervids are natural intermediary hosts of *B. besnoiti* in its heteroxenous life cycle (1, 2, 5–7). Although the definitive host is still unknown, the parasite or its DNA has been isolated from rodents (8), bats (9), felines (10), and foxes (Vulpes vulpes) (11). Transmission may occur by biting insects (e.g., Stomoxys or Tabanus species) or through the use of same needles in different animals (12, 13). Intradermal bradyzoite inoculation revealed a consequent higher clinical score than the subcutaneous or intravenous inoculation (14). Natural mating was also considered to be a significant risk factor for seronegative cows that have contact with positive bulls (15). Cattle that recover from clinical disease or that are asymptomatic remain lifelong carriers and may be a source of infection (2, 16). To the best of our knowledge, there are no reports of human infection.

The first description of cattle infection was in France (17). Bovine besnoitiosis is endemic in southern Europe, eastern Europe, sub-Saharan Africa, and Asia (10, 18–20). Infection in a dairy farm in Ireland was recently reported as the most northerly European described outbreak (21). Given the increasing incidence of bovine besnoitiosis in Europe, it was classified as an emerging disease by the European Food Safety Authority (EFSA) in 2010.

Because the disease's epidemiology is not yet fully understood, it is difficult to establish assertive preventive measures (18). Bovine besnoitiosis is known to be associated with certain climate conditions as high temperature and high precipitation, as well as the abundance of flies and particular seasons. A higher seroprevalence was evidenced at lower latitudes (18, 22, 23).

Two infectious stages of *B. besnoiti* are described and characterized by the infectious form present—tachyzoites or bradyzoites (2). A third stage involving oocysts has been proposed, but its role in the life cycle of the parasite has not been demonstrated yet (24). *Besnoitia besnoiti* presents asexual reproduction mostly in endothelial cells of venous blood vessels (25). It can also proliferate in arterial endothelium, macrophages and fibroblasts particularly in the skin, intermuscular connective tissue, ocular globe, superior respiratory tract, and testicles (3, 26). *Besnoitia besnoiti* cysts were still identified in lungs, vulva, and the parasite DNA was detected in mediastinal lymph nodes, liver, cardiac muscle, ovaries, uterus, masseter muscles, and tonsils (27). Usually, there is a cellular cystic inflammation involving large numbers of macrophages and fewer lymphocytes, granulocytes, eosinophils, and plasmocytes (28). These cysts are often macroscopically visible, and the ones observed in the sclera are considered pathognomonic (18, 29).

The clinical disease is characterized by three successive phases: The first corresponds to a febrile phase; the second is characterized by anasarca; in the last phase, scleroderma and alopecia are predominant (18). However, in most cases, this disease remains subclinical (30). Bulls can develop orchitis, vasculitis, seminiferous tubule degeneration, testicular sclerosis, and atrophy, the disappearance of the germ cells' strata and decreasing number of spermatozoa which frequently results in infertility or even sterility (2, 3, 26). Abortion is common especially in naïve herds. Severely affected animals may die or have to be euthanized for humanitarian reasons (31).

The most common and recommended techniques to diagnose bovine besnoitiosis include indirect fluorescent antibody test (IFAT), enzyme-linked immunosorbent assays (ELISA), Western blot, and skin biopsy—the gold standard (24, 32, 33).

The traditional treatment for clinical cases of bovine besnoitiosis has been the association of an antimicrobial (e.g., oxytetracycline) to a non-steroidal anti-inflammatory and a diuretic (18). The clinical signs (e.g., fever, edema, and anorexia) usually subside a few days after treatment with high doses of intravenous oxytetracycline (Stilwell, personal communication, 2022). Recent *in vitro* studies have reported some potential benefits from certain drugs. Curcumin reduced tachyzoite viability and so may represent a new strategy for bovine besnoitiosis treatment (34). Endochin-like quinolones were considered an outstanding adaptive potential drug, although they showed only an *in vitro* parasitostatic effect on tachyzoite (35). Diclazuril and decoquinate anticoccidials demonstrated an *in vitro* efficacy of 72–90% (36). Despite the possibility of clinical improvement or remission, these animals will most probably remain a source of infection to others (16, 37).

Bovine besnoitiosis has a strong welfare and economic impact on affected animals and farms. Losses are associated with mortality, reduced hide and meat value, male sterility, involuntary culling, and abortion (1, 16).

Precise knowledge on the epidemiology of *B. besnoiti* is extremely important in order to allow for early detection of affected animals and to establish effective and applicable control programs. Thus, the current study aimed to uncover the prevalence of carrier animals in a Portuguese herd and the risk factors associated with the disease, particularly regarding the animals' breed, age, sex, and birthplace.

## 2. Materials and methods

### 2.1. Herd and farm characterization

The study took place in a farm in the south of Lisbon, Portugal. The farm keeps around 2,000 cattle head of four pure breeds—Blonde d'Aquitaine, Charolais, Limousine, and Salers—or their crosses. All suckler herds are permanently on pasture, while weaned and additionally bought-in fattening cattle are raised in outdoor feedlot pens. Most pastures (ryegrass and clover) are irrigated and contiguous although separated by fences.

Vaccination for clostridiosis, bovine viral diarrhea (BVD), infectious bovine rhinotracheitis (IBR), and bovine respiratory syncytial virus (BRSV), as well as deworming, is performed at weaning and then every 6 months. Pregnant animals are also vaccinated at 7 months' gestation, for neonatal diarrhea pathogens (rotavirus, coronavirus, and *Escherichia coli*).

Natural mating is used in the cross-breed herds to produce animals for the feedlot, whereas most cows in the pure breed groups are artificially inseminated. Bulls are with the cows during mating periods and then housed in separate paddocks. Weaning of all calves occurs at ~6 months of age; heifers are first inseminated or put together with the bulls when they are around 18–20 months old or when they have a good body condition (around 65% of mature weight at the commencement of the breeding season).

To improve and increase the genetic quality of the herds, several cows and bulls have been bought in from France, throughout the last decade. These animals are usually kept with the pure breed herds.

### 2.2. Sample collection

Animals were randomly selected for blood sampling when they were brought to the chute for vaccination and/or pregnancy diagnosis. None of the animals had clinical signs compatible with bovine besnoitiosis. Blood was collected into dry tubes by coccygeal venopuncturing with an 18G hypodermic needle. Blood was then refrigerated until centrifugation (2,000 rpm for 10 min) to obtain serum, which was stored in properly identified Eppendorf tubes and frozen at −18°C until sent for laboratory analysis.

A total of 450 animals were sampled and analyzed: 366 of these animals were over 1 year old, and 84 were calves up to 1 year old; 362 were females and 88 were males; as to breed, four were pure Blonde d'Aquitaine, 96 were pure Charolais, 52 were pure Limousin, 69 were pure Salers, and 229 were cross-bred animals.

### 2.3. Laboratory analyses

After thawing, the samples were immediately analyzed. The *Besnoitia besnoiti* isolate used in the preparation of the antigen suspension for the indirect fluorescent antibody test (IFAT) was obtained from a naturally infected bovine (32). Parasites were preserved in liquid nitrogen and thawed for the purposes of this study. Tachyzoites were propagated by continuous passage in Vero cells (African green monkey kidney epithelial cells) were cultured in Dulbecco's Modified Eagle Medium (DMEM) supplemented with penicillin, streptomycin, 1 mM glutamine, and fetal bovine serum (FBS). Inoculation was carried out in monolayers of Vero cells in DMEM supplemented with 10% FBS, in T75 culture vials using 15 × 10^6^ tachyzoites/ml, respectively. Cultured vials were kept at 37°C in a carbon dioxide (CO_2_) incubator with humid atmosphere at 5% CO_2_. After 48 h, the medium of the inoculated vials was replaced with DMEM with 2% FBS. Infected cultures were daily observed under an inverted microscope with phase contrast to allow a growth monitoring of *B. besnoiti*. The supernatant was collected 5 days post-inoculation and tachyzoites were purified on *Whatman CF-11* cellulose columns (38).

The indirect immunofluorescence slides were sensitized as described by Shkap et al. (39). Purified tachyzoites were washed once with PBS, fixed in paraformaldehyde, and cooled on ice for 30 min. After three wash cycles, the parasites were counted in a *Neubauer* chamber and diluted in PBS to 2 × 10^6^ tachyzoites/ml. Drops of 6 μl of antigen suspension were placed on optical microscope slides, with the aid of a multichannel pipette. Each slide had a total of 12 drops, distributed over two lines. The slides were dried at 37°C and fixed in refrigerated acetone at −20°C, for 10 min. Sensitized slides were kept stored at −30°C until their use. On the day of the technique, the slides were removed from the freezer and left at room temperature for ~5–10 min. A circumference was made around each well of the slide, with a blue permanent marker.

The search for anti-*B. besnoiti* antibodies was performed considering a cutoff dilution of 1:256 (39). Several control sera were used: a negative, an intermediate positive, and a strong positive serum. The positive controls belonged to animals with clinical signs of bovine besnoitiosis and positive for histopathology and IFAT. The negative control was obtained from a geographical area with no reported cases of bovine besnoitiosis and was negative to B-MAT test. A 15 μl volume of diluted serum was distributed in each slide well, which were subsequently incubated in a humid chamber at 37°C for 30 min. Slides were then washed in PBS for 10 min and dried with a dryer. After this process, 15 μl of fluorescein isothiocyanate (Serotec AA123F)'s labeled antibovine conjugate, diluted 1:200 in PBS, was applied to each well. This solution also contained *Evans* Blue, in a 1:100 dilution to reduce unspecific background fluorescence. Slides were again incubated in a humid chamber for 30 min and washed as previously mentioned. A mounting medium, composed of a 50/50 glycerol PBS (pH 9.4) mixture was added to the already dry slides. A coverslip was placed over each slide. The results were read using a fluorescence microscope, with a magnification of 400 X. All samples that showed total peripheral fluorescence were considered positive reactions, and those that revealed apical, partial, or absent fluorescence were considered negative.

### 2.4. Serology

The aim of the immunodiagnostic methods used in this study was to highlight the anti-*B. besnoiti* antibodies generated in subclinically infected cattle (18). Serologic tests are performed with total anti-IgG bovine conjugates (40). IFAT demonstrated a sensitivity and specificity of 89.6 and 99.6%, respectively (41). Its high cost and the need for specialized staff are some of the disadvantages (33). However, IFAT confirms the seropositivity of sera diagnosed as “doubtful” when previously submitted to other techniques (40). Through IFAT, antibodies can be detectable 10 days after intravenous parasite inoculation and 22 days after subcutaneous (42).

### 2.5. Statistical analyses

A *p*-value < 0.05 was considered significant. The statistical program *R* (43) was used, and chi-square test was applied to evaluate the association between two qualitative variables and calculate the odds ratio (OR) of one event in relation to another. 95% confidence intervals (CI) were provided. Fisher's exact test was used when the number of cases in a cell was <5, as chi-square test is invalid in this case. Logistic regression was also used to establish a relationship between a dependent variable (seroprevalence) and a set of independent variables (44). The EpiTools program was used for prevalence calculations (45).

## 3. Results

*Besnoitia besnoiti* overall seroprevalence in the sampled population was 16.89%, while for under 1-year-old calves, it was 4.8% and for adults 19.67% (Table 1).

**Table 1:** Number of tested animals and seroprevalence by risk factor, namely age, breed, sex, place of birth, and place of birth of the mother.

**Risk factor**		**Tested**	**Seropositive**
Age	Under 1 year	84	4 (4.8%)
	Over 1 year	366	72 (19.67%)
Breed	Blonde	4	2 (50%)
	Charolais	96	12 (12.5%)
	Limousin	52	8 (15.4%)
	Salers	69	33 (47.8%)
	Crossbreed	229	21 (9.2%)
Sex	Male	88	6 (6.8%)
	Female	362	70 (19.3%)
Place of birth	Farm	371	44 (11.9%)
	Outside farm^#^	78	32 (41%)
Place of birth of the mother^*^	Farm	370	32 (41%)
	France	77	34 (44.2%)

* The difference to 450 animals are those which place of birth was not possible to determine.

# Including eight animals born in France.

**Figure 1:**
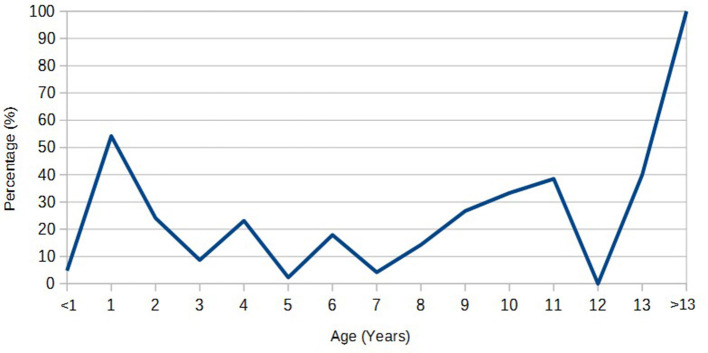
Seroprevalence according to age.

A statistically significant higher seroprevalence was found in older animals (*p* < 0.01) and Salers breed (*p* < 0.01; Table 2). The distribution of the seroprevalence according to age is represented in Figure 1. Regarding sex, we observed 19.3% of seroprevalence in females and only 6.8% in males. However, this variable was not statistically significant (Table 2). Almost half of the animals that were not born in the farm were seropositive for bovine besnoitiosis, while only a 11.9% seroprevalence was observed in animals born at this location (Table 1). Birthplace was considered to be a risk factor, with an OR (95% CI) of 5.14, meaning that the risk of being infected increases ~5 times if the animal was born outside the farm (Table 2).

**Table 2:** Statistical results from the logistic regression: estimate error, significant variables, and odds ratio (95% confidence interval) according to the independent variables sex, place of birth of the mother, age, and breed.

**Estimate error** ***z*** **(Pr** > **|*****z*****|)**					**Odds ratio**
Intercept	−4.4167	0.9307	−4,746	**0.00000208** ^***^	
Sex	−0.5859	0.5460	−1.073	0.283222	**0.56**
Place of birth of the mother	−0.4723	0.6412	−0.737	0.461422	0.62
Age (T > 7)	3.2949	0.7124	4.625	**0.00000375** ^***^	**26.97**
Age (T1–3)	2.3159	0.6108	3.792	**0.000149** ^***^	**10.13**
Age (T3–5)	2.1622	0.6801	3.179	**0.001477** ^**^	**8.69**
Age (T5–7)	2.0682	0.7557	2.737	**0.006204** ^**^	**7.91**
Breed (Blonde)	2.7443	1.3016	2.108	**0.034997** ^*^	**15.55**
Breed (Charolais)	1.6626	0.4668	3.562	**0.000368** ^***^	**2.27**
Breed (Limousine)	0.6831	0.4735	1.443	0.149073	1.98
Breed (Salers)	2.6796	0.7476	3.584	**0.000338** ^***^	**14.58**

Significance: 0^***^; 0.001^**^; 0.01^*^; 0.05; 0.1; 1. Statistically significant values are in bold.

Sampled animals whose mothers were born in Portugal revealed a seroprevalence of 11.4%, whereas those whose mother was born in France (country from which many of the animals were imported) presented a seropositivity of 44.2% (Table 1). In the statistical analysis, the place of birth of the mother was a significant variable (*p* < 0.01) with a 6.14 OR (95% CI; Table 2).

When more than one variable was associated in the logistic regression test, results showed some differences from the ones obtained by chi-square test or *Fisher*'s test. Multivariate analyses revealed that the age between 1 and 3 years or ≥7 years (*p* < 0.01) as well as belonging to Charolais or Salers breeds (*p* < 0.01) were risk factors, while the mother's origin and the sex of the animal lost their significance. Through OR (95% CI) analysis, it was shown that animals ≥7 years are 27 times more likely to be infected and 10 times when aged 1–3 years. Salers breed animals are 14.6 times at higher risk compared with cross-breed animals, and Charolais breed ones are 5.3 times more likely to be infected with *B. besnoiti* (Table 2).

## 4. Discussion

The aim of the study was to determine seroprevalence in large beef herds in Portugal and to assess the risk factors that could be associated with bovine besnoitiosis in a region where the disease might be endemic. This knowledge may prove to be valuable for the development of national and European control programs.

Results highlight age and breed as significant risk factors. Regarding age, our results (3.71 and 21.94% prevalence for calves and adults, respectively) confirm what has been described in literature in which antibodies to *B. besnoiti* are much less prevalent in younger animals (15, 23, 27, 46, 47). Although authors have reported clinical signs in calves younger than 6 months (48, 49), results are in line with the farm records where no animal under 12 months old has ever been diagnosed with clinical bovine besnoitiosis. There are no consensual reasons to explain this apparent resistance to infection in young calves, but there are several theories in literature. The blood-sucking insects, probable vectors of the disease, could be attracted to larger animals because of the major amount of emitted CO_2_ as the animal grows (50, 51). Furthermore, younger animals showed to have higher rates of defensive activities against these arthropods, and hence, the risk of being bitten should be lower (52). There was a peak of seroprevalence in animals from 1 to 2 years of age. This finding is not in line with literature. The observed seropositivity in calves could possibly reflect a passive transmission of antibodies anti-*B. besnoiti* present in the colostrum of infected mothers (53). Maternally derived antibodies were reported to persist in calves up to 6 months of age (54). The mothers of the calves in the study were not determined because blood sampling was aleatory. In a new study, it would be important to identify and test the mothers of positive calves in order to establish a correlation between them. Another hypothesis is that reproduction either by artificial insemination or direct contact through natural mating as risk factors for the transmission of the disease, since the ages at first breeding of cows and bulls were ideally around 18–20 months old. Data on natural mating or artificial insemination were not easily accessed for all animals included in the present study. However, it would be important to include these variables in a future risk factor analysis. Seroprevalence is also higher in animals older than 7 years old. This can be explained by the prolonged and continuous exposure to vectors, as well as by the possibility of transmission by direct contact between animals (e.g., during natural mating or artificial insemination) and the fact that most infected animals are carriers and remain seropositive for life (2, 15, 30), though some cases of serologic remission have been reported (37).

In the 1980's, Salers breed was considered more susceptible to bovine besnoitiosis. Later, this breed susceptibility theory was disputed (28) and abandoned. However, our results showed a significantly higher prevalence of antibodies in Salers (47.8% of tested animals), suggesting that a higher susceptibility may be real for this breed. According to the statistical analyses, Charolais breed may be a risk factor as well.

The ancestors of most seropositive Salers and Charolais had a French origin. Although this might explain the detected higher seroprevalence in these breeds, it does not reject the hypothesis of a genetic predisposition. In the multivariate analyses, the variable “origin of mother” lost its statistical significance, but “breed” remained a risk factor. A recent study demonstrated that the seroprevalence of bovine besnoitiosis in Brown Pyrenean breed was significantly higher as well (47). Further genetic studies are needed in order to confirm whether there is indeed a breed susceptibility to bovine besnoitiosis.

The probabilities of *B. besnoiti* infection in Salers and Charolais breeds are, respectively, 15 and five times greater than in cross-breed animals. Interestingly, cross-breed animals seemed to present a greater resistance to bovine besnoitiosis, independently from age and the dam's breed. All cross-breed subjects were born in Portugal, at the farm. In this context, a partial immune protection might be present in animals that were born in endemic locations, while imported animals could be vulnerable to infection (54). Otherwise, the disease could be imported with the animals, since its diagnosis is not usually carried out during the commercialization of cattle. It would be important in a new study, to unveil Salers and Charolais breed animals' previous history, to better understand how and where they were infected. A common origin or the animals' supplier is an example. A 41% seroprevalence was verified in subjects of external origin, whether Portuguese (from Alentejo, an endemic area) or foreign origin. Once again, these animals might have been imported already infected or the fact that they had been under stress factors, such as transport or vaccination, could have increased their susceptibility to infection at the farm (28). It would be important to clarify whether there is any breed genetic predisposition that would justify a greater vulnerability.

Bovine besnoitiosis economic and welfare severity, the non-remission of the associated protozoa as well as the inexistence of an effective vaccine (55), and the existing lack of information highlight the importance and urgency of an investment in epidemiological investigation as well as diagnosis, treatment, and prophylaxis. In Switzerland, there is already a control program for bovine besnoitiosis (56). Considering the high seroprevalence identified in our study and lately detected in European farms, we believe in the need for the implementation of more rigorous control strategies in the international commercialization of cattle, regarding this neglected disease. The non-acquisition of animals from non-bovine besnoitiosis-free farms and their systematic testing are examples of possible prophylactic measures. Emerging diagnostic techniques for molecular characterization have been suggested (57). A novel highly sensitive and specific ELISA test was recently developed and represents a valuable tool for new epidemiological studies, diagnosis, and the control of bovine besnoitiosis (58). The control of insect vectors is another important measure in endemic locations. This study also suggests that cross-breeding could be a way to prevent or reduce *B. besnoiti* infection as, in our study, these animals demonstrated to be more resistant to the disease.

## 5. Conclusion

Age (1–3 and ≥7 years) and breed (Charolais and Salers) were considered risk factors for *B. besnoiti* infection in this farm. While the first is consensual with many studies, the breed is more controversial as being a risk factor. Further research, including heritability studies and tracing back cattle to their farm of origin, is fundamental to better understand where animals were infected and whether the observed breed predisposition actually indicates genetic susceptibility to Bovine bovine besnoitiosis.

The importation of animals from probable bovine besnoitiosis-free sites to endemic areas could be an important risk factor for the disease.

The high seroprevalence determined in the present study highlights the need for similar studies across southern Europe to establish strong epidemiologic data that would allow the implementation of effective transnational control programs.

There might be a genetic predisposition in these breeds. Thus, genetic studies are needed and should be designed in order to confirm whether there is, indeed, a breed susceptibility to develop bovine besnoitiosis. It would be important, in a new study, to unveil the previous history of Salers and Charolais animals, in order to better understand how and where they were infected.

## Data availability statement

The raw data supporting the conclusions of this article will be made available by the authors, without undue reservation.

## Ethics statement

The animal study was reviewed and approved by Ethic Committee for Research and Teaching (Comissão de Ética para a Investigação e Ensino—CEIE) and Faculty of Veterinary Medicine, University of Lisbon.

## Author contributions

JC: writing—original draft preparation, review and editing, bibliography revision, and investigation. JD: methodology, investigation, laboratory work, and writing—review and editing. HW: laboratory work and writing—review and editing. GS: conceptualization, methodology, writing—review and editing, and supervision. All authors have read and agreed to the published version of the manuscript.
